# Clinical presentation and epidemiological patterns of breast lesions: Experience from a tertiary care hospital

**DOI:** 10.6026/973206300223465

**Published:** 2026-06-30

**Authors:** Chakresh Jain, Sarita Shah, Sarita Singh

**Affiliations:** 1 Department of Community Medicine, Shyam Shah Medical College, Rewa, Madhya Pradesh, India; 2 Department of Obstetrics and Gynecology, Government Medical College, Singrauli, Madhya Pradesh, India; 3 Department of Obstetrics and Gynecology, Shyam Shah Medical College, Rewa, Madhya Pradesh, India

**Keywords:** Breast lesions, epidemiology, breast lump, benign breast disease, breast cancer, tertiary care

## Abstract

Breast lesions constitute a wide spectrum of conditions ranging from benign disorders to malignancies, with varying clinical presentations influenced by demographic and epidemiological factors. Therefore, it is of interest to evaluate the clinical presentation and epidemiological patterns of breast lesions in patients attending a tertiary care hospital. Hence, a prospective observational study was conducted on patients presenting with breast complaints, including detailed clinical evaluation, radiological assessment and histopathological confirmation. The majority of patients presented with breast lumps, with benign lesions being more common in younger age groups and malignant lesions predominating in older patients, showing significant associations with age and symptom duration. Thus, understanding these patterns aids in early diagnosis for appropriate management and improved patient outcomes, especially in resource- limited healthcare settings.

## Background:

Breast lesions represent one of the most common clinical concerns among women worldwide, encompassing a broad spectrum from benign conditions such as fibroadenoma to malignant diseases like carcinoma breast [1]. The epidemiological distribution of these lesions varies significantly across different populations, influenced by factors such as age, reproductive history, lifestyle and access to healthcare services [2]. In recent years, there has been a noticeable increase in the incidence of breast cancer, particularly in developing countries, where late presentation remains a major challenge [3]. Clinically, breast lesions most commonly present as a palpable lump, pain, nipple discharge, or skin changes, with variations in presentation often correlating with the underlying pathology [4]. Early differentiation between benign and malignant lesions is critical, as it directly impacts treatment strategies and prognosis [5]. Despite advancements in imaging and diagnostic techniques, clinical evaluation continues to play a pivotal role in initial assessment, especially in resource-constrained settings [6]. Therefore, it is of interest to analyze the clinical presentation and epidemiological characteristics of breast lesions in patients presenting to a tertiary care hospital, with the aim of improving diagnostic accuracy and guiding effective management strategies.

## Materials and Methods:

This prospective observational study was conducted in the Department of Surgery at a tertiary care hospital over a period of 18 months. All patients presenting with breast-related complaints were evaluated clinically, followed by appropriate radiological investigations (ultrasound/mammography) and histopathological examination wherever indicated.

## Inclusion criteria:

[1] Patients presenting with breast lump, pain, nipple discharge, or other breast-related symptoms

[2] Age ≥ 15 years

[3] Patients willing to participate and provide informed consent

## Exclusion criteria:

[1] Previously diagnosed and treated cases of breast malignancy

[2] Recurrent breast lesions

[3] Patients unwilling to participate

## Statistical analysis:

Data were entered into Microsoft Excel and analyzed using statistical software (SPSS version 24). Continuous variables were expressed as mean ± standard deviation, while categorical variables were presented as frequencies and percentages. Associations between variables were analyzed using the Chi-square test and a p-value < 0.05 was considered statistically significant.

## Results:

A total of 100 patients presenting with various breast-related complaints were included in the study. The clinical and epidemiological characteristics of these patients were analyzed in terms of age distribution, presenting symptoms, laterality of lesions and histopathological findings. The detailed distribution and associations are summarized in the following tables. The majority of patients were in the 26-35 years age group, indicating a higher occurrence of breast lesions among younger females (Table 1). Breast lump was the most common presenting complaint, observed in the majority of cases (Table 2). Left-sided breast involvement was slightly more common compared to the right side (Table 3). Benign lesions, particularly fibroadenoma, constituted the majority of cases (Table 4). Benign lesions were predominant in younger patients, whereas malignant lesions were more common in older age groups (p < 0.05) (Table 5). Malignant lesions were more commonly associated with longer duration of symptoms (>6 months), whereas benign lesions predominated in patients presenting earlier, as illustrated in Figure 1 (see PDF).

## Discussion:

The present study highlights that breast lesions are more commonly observed in younger women, with benign conditions such as fibroadenoma being the predominant pathology, which is consistent with findings reported in previous studies [7]. The higher incidence of breast lumps as the primary presenting symptom aligns with findings reported in similar hospital-based studies [8]. Our findings also demonstrate a significant association between age and the nature of the lesion, with malignant lesions being more frequent in patients above 40 years of age, supporting observations made in recent epidemiological analyses [4]. The slight predominance of left-sided breast involvement noted in this study has also been reported in other studies, although the reason for this remains unclear [9]. The present study also demonstrated that longer duration of symptoms was associated with a higher likelihood of malignancy, as illustrated in Figure 1 (see PDF). The proportion of malignant lesions observed in this study reflects the ongoing challenge of late presentation in developing countries, where awareness and screening programs are still limited [10]. Early detection through clinical examination and appropriate imaging remains crucial for improving outcomes [11]. Early detection through clinical examination, radiological assessment, and histopathological confirmation remains essential for improving prognosis and reducing breast cancer-related morbidity and mortality. The integration of clinical findings with epidemiological characteristics can facilitate risk stratification and support timely intervention. Overall, the present study contributes valuable regional data on the clinicopathological spectrum of breast lesions and reinforces the importance of age-specific diagnostic approaches, early detection strategies, and awareness programs for improving breast health outcomes [12, 13]. Overall, the study emphasizes the importance of correlating clinical findings with epidemiological patterns to facilitate early diagnosis and effective management.

## Conclusion:

Benign breast lesions predominated in younger patients, whereas malignant lesions were more frequently observed in older age groups, with breast lump being the most common clinical presentation. Early clinicopathological evaluation remains essential for accurate diagnosis and timely management of breast lesions. These findings provide valuable epidemiological evidence for improving risk-based assessment and diagnostic decision-making in patients presenting with breast abnormalities.

## Figures and Tables

**Table 1:** Age-wise distribution of patients with breast lesions

**Age Group (years)**	**Number of Patients**	**Percentage (%)**
15-25	28	28%
26-35	32	32%
36-45	18	18%
46-55	12	12%
>55	10	10%

**Table 2:** Distribution of patients according to clinical presentation

**Presentation**	**Number of Patients**	**Percentage (%)**
Breast lump	78	78%
Breast pain	12	12%
Nipple discharge	6	6%
Skin changes	4	4%

**Table 3:** Laterality of breast lesions among study participants

**Side**	**Number of Patients**	**Percentage (%)**
Right	42	42%
Left	50	50%
Bilateral	8	8%

**Table 4:** Histopathological spectrum of breast lesions

**Diagnosis**	**Number of Patients**	**Percentage (%)**
Fibroadenoma	48	48%
Fibrocystic disease	20	20%
Carcinoma	22	22%
Others	10	10%

**Table 5:** Association between age group and nature of breast lesions

**Age Group**	**Benign**	**Malignant**
<40	60	5
≥40	18	17
